# Comparison of the octadentate bifunctional chelator DFO*-*p*Phe-NCS and the clinically used hexadentate bifunctional chelator DFO-*p*Phe-NCS for ^89^Zr-immuno-PET

**DOI:** 10.1007/s00259-016-3499-x

**Published:** 2016-08-30

**Authors:** Danielle J. Vugts, Chris Klaver, Claudia Sewing, Alex J. Poot, Kevin Adamzek, Seraina Huegli, Cristina Mari, Gerard W. M. Visser, Ibai E. Valverde, Gilles Gasser, Thomas L. Mindt, Guus A. M. S. van Dongen

**Affiliations:** 1 Department of Radiology & Nuclear Medicine, VU University Medical Center, De Boelelaan 1085, loc. Radionuclide Center, 1081HV Amsterdam, The Netherlands; 2 Department of Chemistry, University of Zurich, Zurich, Switzerland; 3 Division of Radiopharmaceutical Chemistry, University of Basel Hospital, Basel, Switzerland; 4 ETH Zurich, Institute of Pharmaceutical Sciences, Vladimir-Prelog Weg 4, CH-8093 Zurich, Switzerland; 5 Ludwig Boltzmann Institute for Applied Diagnostics, General Hospital of Vienna, Vienna, Austria

**Keywords:** Immuno-PET, ^89^Zr, DFO*, DFO, Monoclonal antibodies

## Abstract

**Purpose:**

All clinical ^89^Zr-immuno-PET studies are currently performed with the chelator desferrioxamine (DFO). This chelator provides hexadentate coordination to zirconium, leaving two coordination sites available for coordination with, e.g., water molecules, which are relatively labile ligands. The unsaturated coordination of DFO to zirconium has been suggested to result in impaired stability of the complex in vivo and consequently in unwanted bone uptake of ^89^Zr. Aiming at clinical improvements, we report here on a bifunctional isothiocyanate variant of the octadentate chelator DFO* and the in vitro and in vivo comparison of its ^89^Zr-DFO*-mAb complex with ^89^Zr-DFO-mAb.

**Methods:**

The bifunctional chelator DFO*-*p*Phe-NCS was prepared from previously reported DFO* and *p*-phenylenediisothiocyanate. Subsequently, trastuzumab was conjugated with either DFO*-*p*Phe-NCS or commercial DFO-*p*Phe-NCS and radiolabeled with Zr-89 according to published procedures. In vitro stability experiments were carried out in saline, a histidine/sucrose buffer, and blood serum. The in vivo performance of the chelators was compared in N87 tumor-bearing mice by biodistribution studies and PET imaging.

**Results:**

In 0.9 % NaCl ^89^Zr-DFO*-trastuzumab was more stable than ^89^Zr-DFO-trastuzumab; after 72 h incubation at 2-8 °C 95 % and 58 % intact tracer were left, respectively, while in a histidine-sucrose buffer no difference was observed, both products were ≥ 92 % intact. In vivo uptake at 144 h post injection (p.i.) in tumors, blood, and most normal organs was similar for both conjugates, except for skin, liver, spleen, ileum, and bone. Tumor uptake was 32.59 ± 11.95 and 29.06 ± 8.66 % ID/g for ^89^Zr-DFO*-trastuzumab and ^89^Zr-DFO-trastuzumab, respectively. The bone uptake was significantly lower for ^89^Zr-DFO*-trastuzumab compared to ^89^Zr-DFO-trastuzumab. At 144 h p.i. for ^89^Zr-DFO*-trastuzumab and ^89^Zr-DFO-trastuzumab, the uptake in sternum was 0.92 ± 0.16 and 3.33 ± 0.32 % ID/g, in femur 0.78 ± 0.11 and 3.85, ± 0.80 and in knee 1.38 ± 0.23 and 8.20 ± 2.94 % ID/g, respectively. The uptake in bone decreased from 24 h to 144 h p.i. about two fold for the DFO* conjugate, while it increased about two fold for the DFO conjugate.

**Conclusions:**

Zr-DFO*-trastuzumab showed superior in vitro stability and in vivo performance when compared to ^89^Zr-DFO-trastuzumab. This makes the new octadentate DFO* chelator a candidate successor of DFO for future clinical ^89^Zr-immuno-PET.

**Electronic supplementary material:**

The online version of this article (doi:10.1007/s00259-016-3499-x) contains supplementary material, which is available to authorised users.

## Introduction

PET imaging with ^89^Zr-labeled monoclonal antibodies (mAbs) or other targeted vehicles (e.g., peptides, nanoparticles, and cells), collectively called ^89^Zr-immuno-PET, can be used for better understanding of disease targets and the in vivo behavior of targeted drugs [1]. ^89^Zr is ideally suited for this purpose because its physical half-life of 78.4 h matches the residence time of intact mAbs in the body (typically several days). Moreover, ^89^Zr is a residualising radionuclide; when a mAb internalises, ^89^Zr-mAbs give higher tumor-to-normal tissue ratios than, e.g., the corresponding ^124^I-labeled mAbs [2] resulting in better contrast on PET images.

The field of ^89^Zr-immuno-PET is expanding very rapidly as exemplified by the increasing number of publications in recent years, the types of applications, and the number of clinical trials [1, 3]. Several factors are contributing to this rapid expansion. First, mAbs are entering the mainstream of targeted drug development and therapy [4]. The recent introduction of the next generation of mAbs, characterised by increased potency (e.g., antibody-drug conjugates and immune checkpoint inhibiting mAbs), multiple binding domains, and/or high costs, makes finding answers about their in vivo behavior in individual subjects even more urgent [5]. Second, ^89^Zr-immuno-PET appears to be ideally suited for quantitative imaging of mAbs, and became technically matured by the commercial supply of ^89^Zr and chelators, standard protocols for ^89^Zr coupling to mAbs, and standardisation and harmonisation of ^89^Zr-quantification by PET imaging [6–8].

The only chelator used thus far in the clinic for the complexation of ^89^Zr is desferrioxamine (DFO). The fact that DFO was clinically used for many years to counteract iron and aluminum overload, has clearly facilitated the introduction of DFO in clinical ^89^Zr-immuno-PET. Moreover, the first clinical ^89^Zr-immuno-PET trial indicated that the chelator is not immunogenic [9], and can, therefore, be used repeatedly. The most often used conjugation procedures employ a 2,3,5,6-tetrafluorophenol (TFP) activated ester of *N*-succinyl-DFO-Fe (TFP-*N*-suc-DFO-Fe, in which DFO is protected by Fe^3+^) or *p*-isothiocyanatobenzyl-DFO (*p*-SCN-Bn-DFO = DFO-*p*Phe-NCS, Macrocyclics), forming stable amide or thiourea bonds, respectively, with lysine residues of the proteins [6, 10–12]. Both bifunctional chelating agents are commercially available and have been applied in clinical trials.

From an inorganic chemistry perspective, however, DFO is not the optimal chelator for stable complexation of zirconium, because DFO consists of three hydroxamate moieties and is a hexadentate chelator, while ^89^Zr^4+^ prefers the formation of octadentate complexes [13]. Although the resulting slightly impaired stability of ^89^Zr-DFO-mAb does not really hamper image quality, it is clear from preclinical studies that in time, some ^89^Zr^4+^ becomes released from the conjugate and accumulates in the bones [13–16]. For example, Perk et al. compared the biodistribution of the conjugates ^89^Zr-DFO-cetuximab, ^88^Y-DOTA-cetuximab and ^177^Lu-DOTA-cetuximab in tumor-bearing nude mice [14]. While the overall biodistribution of these conjugates was very similar, a significant higher uptake for ^89^Zr compared to ^88^Y and ^177^Lu was observed in femur and sternum at 48–144 h p.i. Over time, femur levels increased from about 2 %ID/g at 24 h p.i. to 6.9 %ID/g at 144 h p.i., indicating partial instability of the ^89^Zr-DFO complex. Bone uptake of released ^89^Zr is unwanted, since it will increase the radiation dose to the bone marrow, impair detection of bone metastases, and disturb ^89^Zr-mAb quantification in bone, bone marrow, and bone metastases.

An optimal chelating agent should be safe for clinical use, efficiently conjugated to, e.g., mAbs and labeled with ^89^Zr, not alter the biodistribution of the mAb, and, most importantly, be more stable in vitro and in vivo than DFO to prevent gradual accumulation of ^89^Zr in the bones. In the past years, several new chelators have been reported aiming at increased ^89^Zr-complex stability in vivo. Some chelators contain hydroxamate moieties, like the linear and macrocyclic tetrahydroxamate chelators reported by Guerard et al., and the trihydroxamate chelators reported by Boros et al. and Zhai et al. [17–20]. Other examples do not contain hydroxamate moieties like *p*-SCN-Bn-H6phospha, 3,4,3-(LI-1,2-HOPO), and 3-hydroxypyridin-2-one (2,3-HOPO) [16, 21–23]. However, none of these developments have led to a bifunctional chelator that outperforms ^89^Zr-DFO-mAb complexes on all abovementioned aspects of an optimal chelating agent.

Recently, we reported the synthesis of a tetrahydroxamate chelator called DFO* (DFO-star), and the high stability of its ^89^Zr-DFO*-complex as shown in challenging experiments with DFO [24]. In the current paper, we describe the synthesis of the bifunctional DFO*-*p*Phe-NCS, its coupling to mAbs, and the subsequent labeling with ^89^Zr. Finally, ^89^Zr-DFO*-mAb conjugates are compared with ^89^Zr-DFO-mAb conjugates for stability in vitro, and for biodistribution and PET imaging in vivo in tumor-bearing mice with emphasis on ^89^Zr bone uptake.

## Materials and methods

Details regarding the synthesis and analysis of DFO*-pPhe-NCS, radiolabeling results of cetuximab and rituximab, and examples of SEC-HPLC diagrams of ^89^Zr-DFO*-trastuzumab, ^89^Zr-DFO*-rituximab, and ^89^Zr-DFO*-cetuximab can be found in the supplemental data.

### Materials, monoclonal antibodies, cell lines, radioactivity, and nomenclature

All reagents and solvents were purchased from Sigma Aldrich. Trastuzumab (21 mg/mL) directed against human epidermal growth factor receptor 2 (HER2), cetuximab (5 mg/mL) directed against the epidermal growth factor receptor (EGFR), and rituximab (10 mg/mL) directed against CD20, were obtained from the VU University Medical Center pharmacy. In the present study, DFO*-*p*Phe-NCS is compared with DFO-*p*Phe-NCS, which is commercially available from Macrocyclics under the name *p*-SCN-Bn-deferoxamine. Both compounds have the same *p*-isothiocyanatophenylurea linker attached to DFO or DFO*, respectively, and will be designated ^89^Zr-DFO(*)-mAb throughout the manuscript. The human gastric cancer cell line NCI-N87 was obtained from ATCC (United Kingdom) after cytogenetic testing and used within 6 months after resuscitation of the frozen cell line. The immunoreactivity was determined using SKOV-3 (HER2), A431 (EGFR), or SU-DHL-4 (CD20) cells essentially as described by Lindmo et al. [25]. ^89^Zr (≥ 0.15 GBq/nmol in 1 mol/L oxalic acid) was obtained from Perkin Elmer, Boston, USA.

#### General analyses and procedures

Cetuximab (5 mg/mL) was rebuffered to 0.9 % NaCl with the aid of size exclusion chromatography using PD10 columns (GE Healthcare Life Sciences) and re-concentrated to 5 or 5.77 mg/mL by a spin filter (Microcon-10 centrifugal filter, Merck Millipore). Trastuzumab (21 mg/mL) and rituximab (10 mg/mL) were used as such and diluted to 5 or 5.77 mg/mL with 0.9 % NaCl.

Nanodrop analyses were performed on a NanoVue Plus (GE Healthcare Life Sciences). Spin filter separation was performed to determine the radiochemical purity. To this end 4 μL of sample was diluted with 96 μL eluent (5 % DMSO and 95 % 20 mM histidine/240 mM sucrose buffer pH 5.5–5.8) and applied on a microcon-30 centrifugal filter unit (Ultracel YM-30, regenerated cellulose, 30 kDa cut-off, Merck Millipore). The solution was spun down for 7 min at 14,000 rpm (Eppendorf 5430). The filter was washed twice with 100 μL eluent and spun down at 14,000 rpm for 7 min after each wash step. The filtrate contained free ^89^Zr/^89^Zr-DFO(*), while the radiolabeled mAb was left on the filter. Size exclusion (SE)-HPLC was performed to determine protein integrity and radiochemical purity. SE-chromatography was performed on a Jasco HPLC system equipped with a superdex^TM^ 200 10/30 GL size exclusion column (GE Healthcare Life Sciences) or Zenix SEC-300 (3 μm, 300 Å, 7.8x50 mm, Sepax) column including a guard column using a mixture of 0.05 M sodium phosphate, 0.15 M sodium chloride (pH 6.8), and 0.01 M NaN_3_ as the eluent at a flow rate of 0.5 mL/min or 1 mL/min, respectively. The radioactivity of the eluate was monitored using an inline NaI(Tl) radiodetector (Raytest Sockett). HPLC monitoring of the final products was performed on a Jasco HPLC system using a superdex 200 column. Serum stability samples were analysed on a Zenix SEC-300 using the same eluent as the superdex 200 column. The iTLC analysis was performed to determine the radiolabeling efficiency of DFO*-*p*Phe-NCS and DFO-*p*Phe-NCS with ^89^Zr using iTLC-SG (Agilent Technologies, Santa Clara, CA, USA) and 50 mM DTPA pH = 7 as mobile phase (11.5 cm strip, 8 min run time). ^89^Zr-DFO*-*p*Phe-NCS had a R_f_ = 0–0.25, ^89^Zr-DFO-*p*Phe-NCS a R_f_ = 0, and free ^89^Zr a R_f_ = 1.

#### Preparation of ^89^Zr-DFO*-mAb and ^89^Zr-DFO-mAb

The pH of the mAb solution (5 mg/mL) was adjusted to 8.9-9.1 with 0.1 M Na_2_CO_3_. This solution was added to 3 eq. of DFO*-pPhe-NCS or DFO-pPhe-NCS in DMSO (2 v/v% compared to aqueous solution) and incubated for 30 min in a Thermomixer (550 rpm) at 37 °C. After 30 min, non-conjugated hydrolysed chelator was removed by size exclusion chromatography using a PD-10 column and 0.9 % NaCl or 20 mM histidine + 240 mM sucrose pH 5.5–5.8 as eluent (same eluent used as after radiolabeling). The concentration of the product was determined by spectrophotometry at 280 nm using NanoDrop or by a calibration curve on a superdex 200 column. Subsequently, DFO*-mAb or DFO-mAb was radiolabeled with ^89^Zr at room temperature for 60 min. Typically, for a 1 mL reaction the following protocol was used: to 100 μL ^89^Zr (∼50 MBq) in 1 M oxalic acid solution, 45 μL 2 M Na_2_CO_3_ was added and reacted for 3 min. Subsequently, 150 μL 0.5 M HEPES buffer (pH 7.0), 355 μL (0.5 mg) DFO*-mAb, or DFO-mAb and 350 μL 0.5 M HEPES (pH 7.0) were added (the second portion of HEPES may be added with the first portion, as long as the reaction is properly mixed after the addition of mAb). Finally, ^89^Zr-DFO*-mAb or ^89^Zr-DFO-mAb was purified by size exclusion chromatography (PD10 column). The concentration of DFO(*)-mAbs was determined by SE-HPLC on a Zenix SEC-300 column. For in vitro stability experiments with trastuzumab, the product was formulated to 0.2 mg/mL and 10 MBq/mL (50 MBq/mg) (see in vitro stability section for buffer composition).

#### Chelator-to-mAb ratio of ^89^Zr-DFO*-mAb and ^89^Zr-DFO-mAb

The chelator-to-mAb ratio was determined by prelabeling the chelator and the subsequent conjugation of ^89^Zr-chelator as well as by an isotopic dilution assay essentially as described by Meares et al. [26].

Prelabeling the bifunctional chelator: to 20 μL ^89^Zr (2–5 MBq) in 1 M oxalic acid solution, 100 μL 0.9 % NaCl and 9 μL 2 M Na_2_CO_3_ were added and reacted for 3 min. Subsequently, 100 μL 0.5 M HEPES buffer (pH 7.0) and 40 μL DFO*/DFO-*p*Phe-NCS (5 mM) in DMSO were added. After 5 min, half of the volume (134.5 μL) was transferred to an Eppendorf vial, and to this solution 865.5 μL trastuzumab, rituximab or cetuximab (5.77 mg/mL) were added (final concentration: 5 mg/mL mAb). The pH was set to 8.9-9.1 with 2 M Na_2_CO_3,_ and the reaction incubated for 30 min in a Thermomixer (550 rpm) at 37 °C. Finally, ^89^Zr-DFO*-mAb/^89^Zr-DFO-mAb was purified by size exclusion chromatography (PD10 column) and the conjugation efficiency determined.

Isotopic dilution assay: the DFO(*)-to-trastuzumab and DFO(*)-to-rituximab molar ratios were determined following a general method as described by Meares et al. [26]. In short, conjugates were labelled according to the aforementioned procedure with a known excess of zirconium oxalate spiked with ^89^Zr.

#### In vitro stability (aqueous solutions and blood serum)

The in vitro stability was evaluated in two sets of experiments. In a first set of experiments, the products were formulated at 0.2 mg/mL and 10 MBq/mL (50 MBq/mg) and stored at 4 °C and room temperature in two different solutions: 0.9 % NaCl or 20 mM histidine + 240 mM sucrose, pH 5.5-5.8. After 24, 48, 72, and 168 h samples were analysed for radiochemical purity by a spin filter and immunoreactivity by a Lindmo assay. In a second set of experiments, the formulated products in 0.9 % NaCl was mixed with freshly prepared human blood serum at a 1:1 ratio, and 0.02 % sodium azide was added. The samples were incubated at 37 °C in a CO_2_-enriched atmosphere (5 % CO_2_). At various time points, aliquots were taken and analysed for radiochemical purity by SE-HPLC on a Zenix SEC-300 column and for immunoreactivity by a Lindmo assay. In the case of serum, SE-HPLC was used for analysis of radiochemical purity, because spin filters gave less reproducible results.

#### Evaluation of in vivo biodistribution

The biodistribution of both ^89^Zr-DFO*-trastuzumab and ^89^Zr-DFO-trastuzumab was evaluated in N87 tumor bearing mice. Female mice (HSD:Athymic Nude-Foxn1nu, 21–31 g; Harlan) were 8 to 10 weeks old at the time of the experiments. All animal experiments were done according to the NIH Principles of Laboratory Animal Care and Dutch national law (“Wet op de dierproeven”, Stb 1985, 336). Mice bearing N87 xenografts (two groups of *n* = 18) were anesthetised by inhalation of 2 % isoflurane and injected intravenously (i.v.) via the retroorbital plexus with either 100 μg ^89^Zr-DFO-trastuzumab or ^89^Zr-DFO*-trastuzumab (2 MBq) in 20 mM histidine + 240 mM sucrose, pH 5.5-5.8. Unlabeled trastuzumab was added to the injection mixture to bring the total mAb dose to 100 μg per mouse. At 1, 4, 24, 48, 72, and 144 h p.i., blood samples were drawn to determine the blood kinetics. At 24, 72, and 144 h p.i., six mice of each group were anesthetised, bled, killed, and dissected. After blood, tumor, and normal tissues had been weighed, the amount of radioactivity in each sample was measured in a gamma counter. Radioactivity uptake was calculated as the percentage of the injected dose per gram of tissue (%ID/g).

#### PET study

PET imaging was performed on a dedicated small animal NanoPET/CT scanner (Mediso Ltd., Hungary, Szanda et al.). N87 xenograft-bearing nude mice (*n* = 1 for each conjugate) from the 144 h biodistribution timepoint were anesthetised by inhalation of 2 % isoflurane and scanned at 72 h p.i. for 1 h. A CT scan was acquired prior to the PET scan and used for attenuation and scatter correction purposes. Reconstruction was performed with a fully 3-dimensional (3-D) reconstruction (Tera-Tomo; Mediso Ltd.) with four iterations and six subsets, resulting in an isotropic 0.4 mm voxel dimension. The scanner was cross-calibrated with the dose calibrator and well counter, enabling the derivation of accurate SUV measures.

#### Statistical analyses

Statistical analysis was performed on pharmacokinetics, tissue uptake, and tumor-to-blood ratios between different groups of mice with the Welch’s *T*-test (SPSS) for paired data. Two-sided significance levels were calculated and *p* < 0.01 was considered as statistically significant.

## Results

**Fig. 1:**
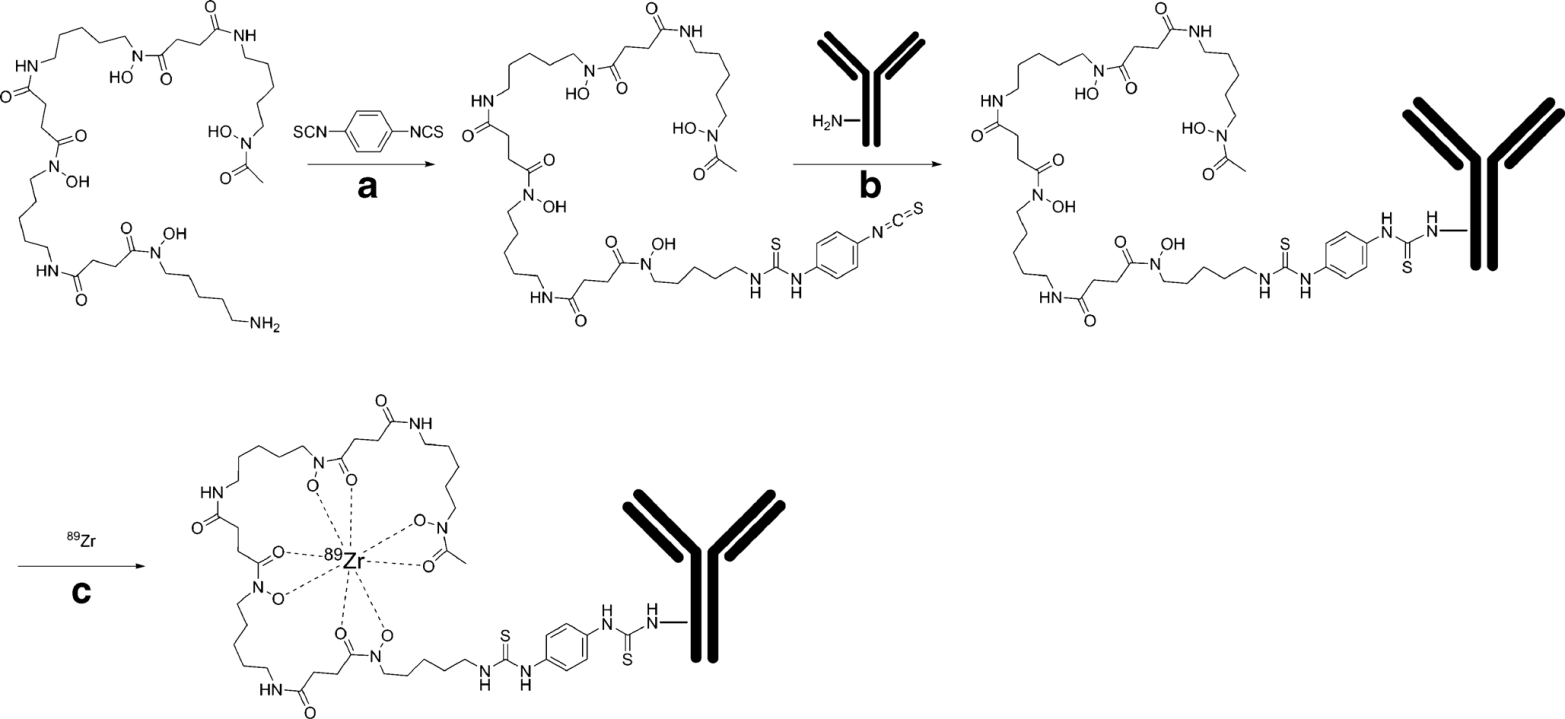
Synthesis of ^89^Zr-DFO*-mAbs: **a** Et_3_N, *i*PropOH/H_2_O/CHCl_3_, RT, 18 h; **b** 30 min 37 °C, RT; **c** 60 min RT

DFO*-*p*Phe-NCS was prepared from DFO* [24] in a single step by reaction with *p*-phenylenediisothiocyanate (Fig. 1). The product was purified by preparative HPLC and isolated in a satisfying chemical yield with a purity of >95 % according to HPLC at 275 nm and NMR (Figure S1-4). For conjugation to mAbs, the product was dissolved in DMSO.

^89^Zr-DFO*-trastuzumab and ^89^Zr-DFO-trastuzumab were prepared similarly to postlabeling procedures described by Vosjan et al. [6]. Protein aggregation during modification was observed when using the Vosjan method, which could be minimised by adapting the reagent addition procedure. Instead of adding small portions of the chelator in DMSO to the mAb solution, the mAb was added at once to the chelator in DMSO. This way, the protein integrity was preserved, showing a single IgG peak at 280 nm at SE-HPLC. All conjugates could be radiolabeled efficiently (>80 % radiolabeling yield with 0.5 mg/mL mAb) and had a radiochemical purity of >97.5 % and optimal immunoreactive fraction (>96 %). This is also true for cetuximab and rituximab, which could be radiolabeled with the same efficiency and radiochemical purity and an immunoreactive fraction ≥90 % (see supporting information).

The DFO to mAb molar ratios as determined by the isotope dilution assay were 0.9 ± 0.1 (*n* = 6) and 1.3 ± 0.1 (*n* = 3) for trastuzumab and rituximab, respectively, and the DFO* to mAb ratios were 0.6 ± 0.1 (*n* = 4) and 0.8 ± 0.1 (*n* = 4) for trastuzumab and rituximab, respectively.

Interestingly, when DFO*-pPhe-NCS and DFO-pPhe-NCS were first radiolabeled with ^89^Zr and subsequently conjugated to the mAb (prelabeling approach), only for DFO* radiolabeled mAb was obtained. Although DFO*-*p*Phe-NCS and DFO-*p*Phe-NCS did radiolabel efficiently (after 5 min at room temperature both compounds were >95 % radiolabeled as determined by iTLC), ^89^Zr-DFO-*p*Phe-NCS did not conjugate to mAbs at all. In contrast, in the case of DFO*, the applied 3:1 conjugation ratio of ^89^Zr-DFO*-*p*Phe-NCS to mAb resulted in 43 ± 2, 63 ± 1 and 43 ± 2 % radiolabeled product and, thus, a 1.3, 1.9, and 1.3 chelator-to-mAb ratio for trastuzumab (*n* = 4), rituximab (*n* = 2), and cetuximab (*n* = 2), respectively.

^89^Zr-DFO*-trastuzumab and ^89^Zr-DFO-trastuzumab obtained by standard post radiolabeling procedures were stored at 4 °C and room temperature in 0.9 % NaCl and 20 mM histidine/240 mM sucrose pH 5.5-5.8 up to 7 days to evaluate the in vitro stability. In 0.9 % NaCl, ^89^Zr-DFO*-trastuzumab outperformed ^89^Zr-DFO-trastuzumab in terms of stability (see Table 1 and Table S1); while at 4 °C only 57.6 % intact ^89^Zr-DFO-trastuzumab was left after 3 days, ^89^Zr-DFO*-trastuzumab was still 94.5 % intact after the same period of time. At room temperature, the percentage of intact tracer was 41.1 % for ^89^Zr-DFO-trastuzumab and 90.7 % for ^89^Zr-DFO*-trastuzumab. In 20 mM histidine/240 mM sucrose, the products exhibited no difference in stability. Both compounds were >92 % intact after 3 days of storage at 4 °C or room temperature. The immunoreactivity of the products, not corrected for free radioactivity, decreased accordingly and was following the same trend as the radiochemical purity. Also in vitro stability studies in human serum/0.9 % NaCl (1:1) showed that ^89^Zr-DFO*-trastuzumab is more stable than ^89^Zr-DFO-trastuzumab. After 72 h at 37 °C, 97.5 % and 77.7 % intact tracer was left, respectively. Immunoreactivity determined by the Lindmo binding assay showed the same trend and an immunoreactive fraction of 91 % and 74 % was observed after 72 h at 37 °C for ^89^Zr-DFO*-trastuzumab and ^89^Zr-DFO-trastuzumab, respectively.

**Table 1:** In vitro stability of ^89^Zr-DFO*-trastuzumab (A) and ^89^Zr-DFO-trastuzumab (B) at 4 °C in 20 mM histidine + 240 mM sucrose or 0.9 % NaCl or at 37 °C in serum

A	20 mM Histidine/240 mM sucrose		0.9 % NaCl		Serum	
	4 °C		4 °C		37 °C	
	Radiochemical purity (%)	Immunoreactive fraction (%)	Radiochemical purity (%)	Immunoreactive fraction (%)	Radiochemical purity (%)	Immunoreactive fraction (%)
0 h	99.0	98	98.0	97	100.0	97
24 h	98.4	97	97.8	95	100.0	nd
48 h	97.7	96	95.1	93	99.2	nd
72 h	97.0	94	94.5	91	97.5	91
168 h	92.5	90	89.1	82	96.3	88
B	20 mM Histidine/240 mM sucrose		0.9 % NaCl		Serum	
	4 °C		4 °C		37 °C	
	Radiochemical purity (%)	Immunoreactive fraction (%)	Radiochemical purity (%)	Immunoreactive fraction (%)	Radiochemical purity (%)	Immunoreactive fraction (%)
0 h	97.8	96	98.6	97	100.0	97
24 h	97.2	95	88.5	81	88.6	85
48 h	96.0	93	65.8	nd	82.3	79
72 h	94.9	93	57.6	nd	77.7	74
168 h	90.5	87	50.4	nd	72.0	64

*nd* not determined

Radiochemical purity of buffer samples determined by a spin filter

Radiochemical purity of serum samples determined by SEC-HPLC

**Fig. 2:**
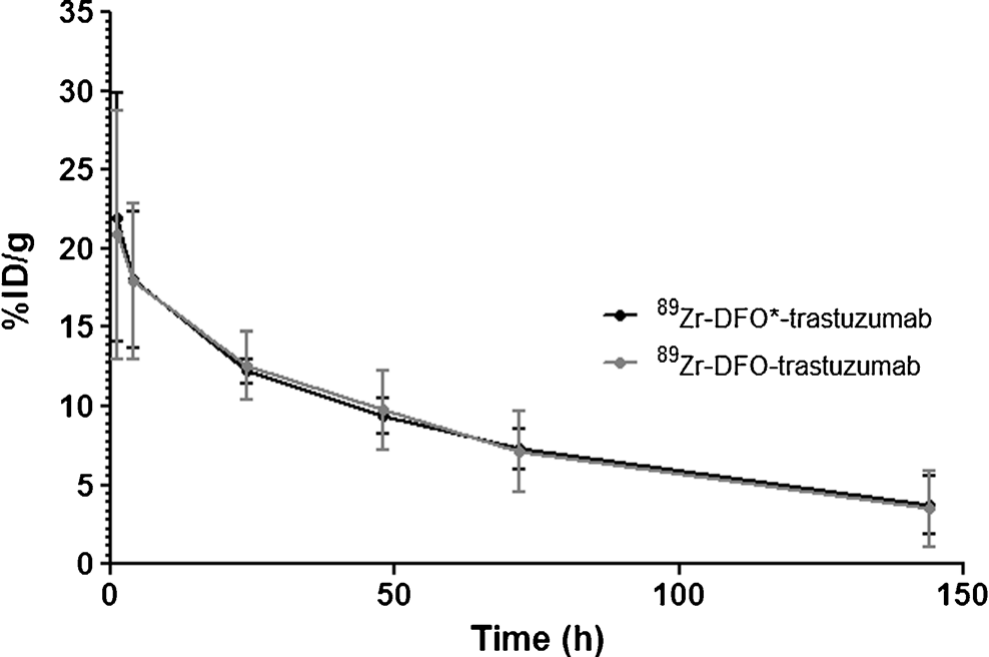
Blood kinetics of ^89^Zr-DFO*-trastuzumab (*black line*) and ^89^Zr-DFO-trastuzumab (*grey line*) in N87 tumor-bearing nude mice up to 144 h after administration

**Fig. 3:**
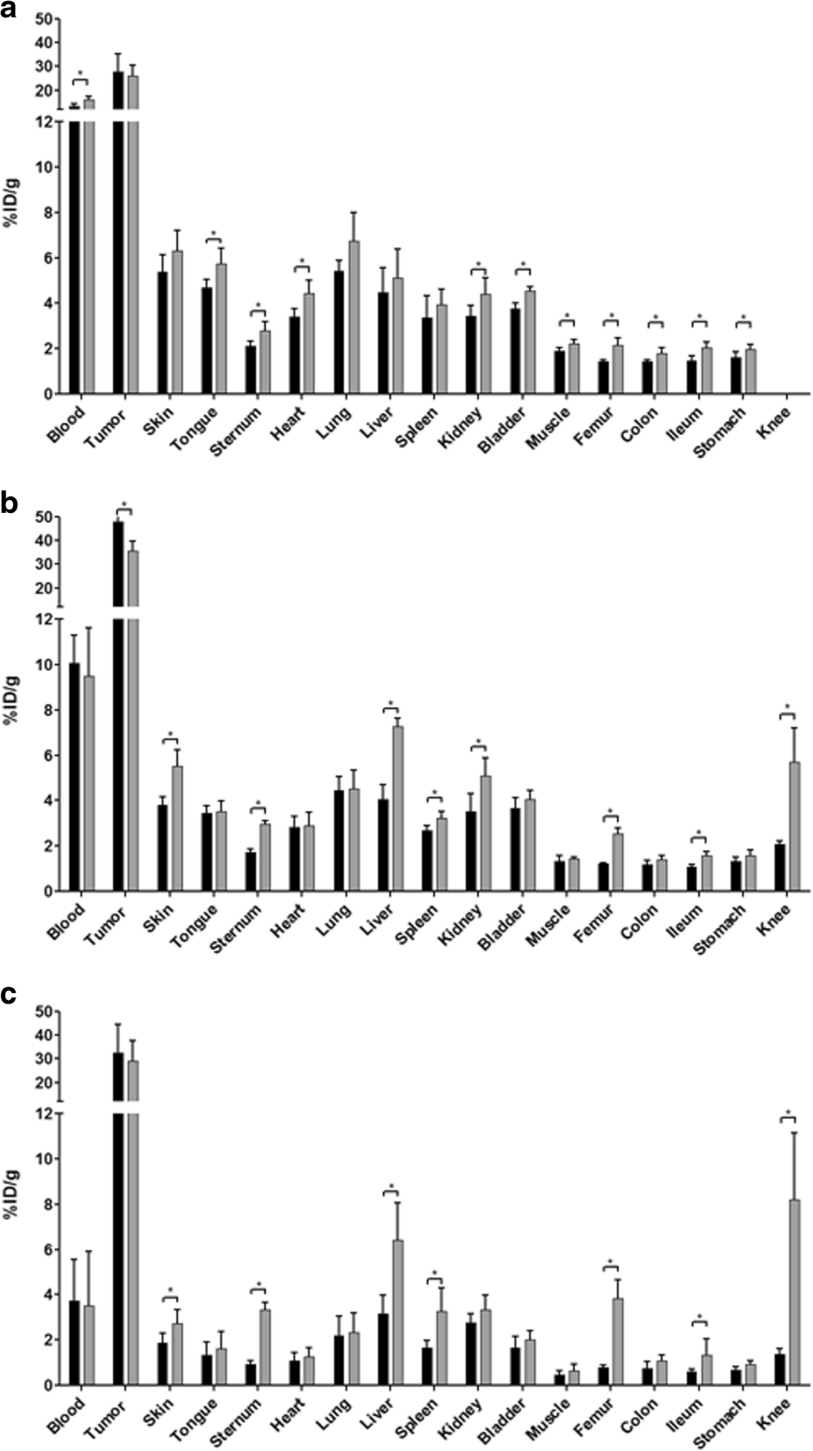
Biodistribution of ^89^Zr-DFO*-trastuzumab (*black bars*) and ^89^Zr-DFO-trastuzumab (*grey bars*) in N87 tumor-bearing nude mice at 24 h **a**, 72 h **b**, and 144 h **c** after administration. Total administered dose 100 μg. Mean (%ID/g) ± SD at each time point after injection (*n* = six animals per time point per conjugate). (Significant differences (*P* < 0.05) in biodistribution between both radioimmunoconjugates are marked with an *asterisk*)

^89^Zr-DFO*-trastuzumab and ^89^Zr-DFO-trastuzumab were injected into N87 tumor-bearing nude mice. At 1, 4, 24, 48, 72, and 144 h p.i., blood samples were drawn and the % ID/g determined. At 24, 72, and 144 h p.i., the %ID/g uptake in tumor and normal tissue was determined. Blood kinetics were similar (see Fig. 2). At 144 h p.i., blood kinetics and tumor uptake were not significantly different, while bone-containing organs, skin, liver, spleen, and ileum showed a significantly lower uptake level in case of DFO* (see Fig. 3). Uptake of ^89^Zr-DFO*-trastuzumab in bone-containing organs decreased over time, while the uptake of ^89^Zr-DFO-trastuzumab increased (see Figure S5). As a result, ratios of ^89^Zr-DFO-trastuzumab over ^89^Zr-DFO*-trastuzumab in bone-containing organs increased over time (see Figure S6). At 24 h p.i., the ratio was 1.3 in sternum and 1.5 in femur; at 72 h p.i. 1.7 in sternum, 2.1 in femur, and 2.8 in knee; and at 144 h p.i., 3.6 in sternum, 5.0 in femur, and 5.9 in knee (see Figure S6).

**Fig. 4:**
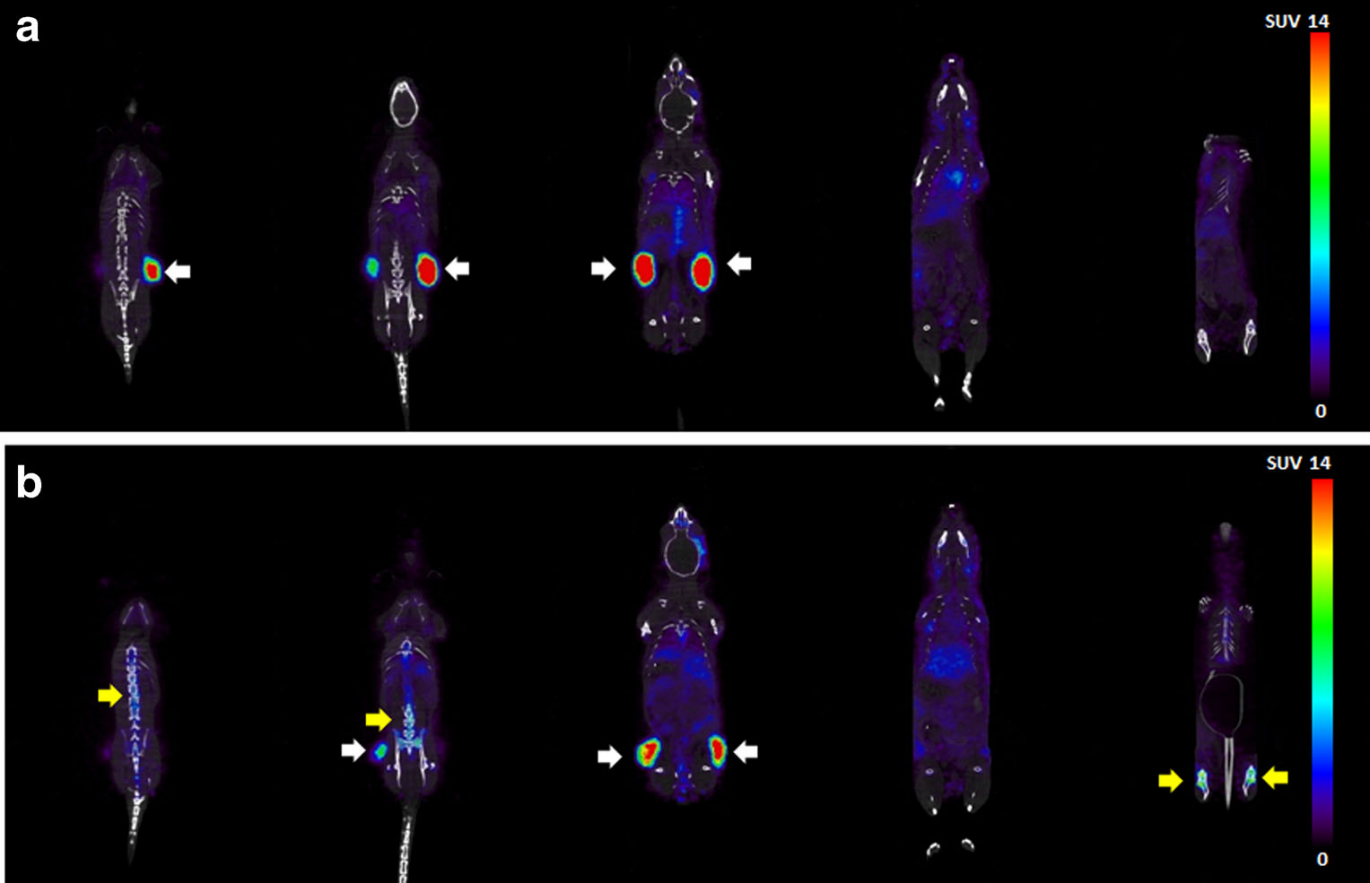
Coronal PET images of N87 tumor-bearing nude mice acquired 72 h after injection of 100 μg, 2 MBq of either ^89^Zr-DFO*-trastuzumab **a** or ^89^Zr-DFO-trastuzumab **b**.Tumor are indicated with *white arrows*, bone uptake is indicated with *yellow arrows*

PET imaging studies were performed to evaluate ^89^Zr-DFO(*)-trastuzumab uptake also in tissues not evaluated in the ex vivo biodistribution experiment. Movies of PET images that were obtained 72 h p.i. are provided in the supporting information. While tumor uptake was clearly visible for both ^89^Zr-DFO*-trastuzumab and ^89^Zr-DFO-trastuzumab, ^89^Zr-DFO*-trastuzumab showed less bone uptake than ^89^Zr-DFO-trastuzumab (see Fig. 4).

## Discussion

In the present study, we describe a new bifunctional octadentate chelator for ^89^Zr, which holds great promise to improve clinical ^89^Zr-immuno-PET performance.

Previously, we reported the synthesis of DFO* and the superior in vitro stability of its complex with ^89^Zr in comparison to ^89^Zr-DFO, also when challenged with an excess of DFO [24]. Here, we describe the synthesis of a bifunctional version of this chelator, DFO*-*p*Phe-NCS. This bifunctional chelator is prepared starting from the commercially available DFO, which makes DFO*-based chelating agents attractive candidates for commercialization and wide spread use in clinical nuclear medicine. The GMP compliant synthesis of ^89^Zr-DFO-mAb conjugates has been described before [6] and ^89^Zr-DFO*-mAbs are prepared in a similar manner. The only difference is a small adjustment of the conjugation protocol, which resulted in improved integrity for the ^89^Zr-DFO(*)-mAb conjugates. The addition of small portions of the chelator in DMSO to mAbs can result in protein aggregation. To prevent this, the addition order was reversed, and the protein added to the chelator instead. In this way protein aggregation could be minimised. The conjugation of DFO*-*p*Phe-NCS was slightly less effective, but the subsequent radiolabeling efficiency was similar for both chelators. For DFO-*p*Phe-NCS, the same chelator-to-mAb ratios and radiolabeling efficiencies were achieved as reported by Vosjan et al. [6]. Using a prelabeling approach, we found that ^89^Zr-DFO-*p*Phe-NCS could not be conjugated to mAbs anymore, while ^89^Zr-DFO*-*p*Phe-NCS was still capable of conjugation. This indicates that the isothiocyanate linker may be coordinated to ^89^Zr in case of DFO and, consequently, is not available anymore for bioconjugation. The same accounts for the *N*-suc-DFO chelator. When *N*-suc-DFO was first radiolabeled with Zr-89, the COOH group could not be transformed into the TFP-ester anymore [10]. In the case of DFO*; however, ^89^Zr is coordinatively saturated by the chelator, and; therefore, the isothiocyanate linker is left available for bioconjugation. This interesting difference now allows prelabeling strategies with ^89^Zr using DFO*, which was not possible before with DFO derivatives. Prelabeling with ^89^Zr-DFO*-*p*Phe-NCS could be an advantageous approach in the case of labeling of small amounts of mAb, cells [27], or mAbs with supertoxic payloads, since the number of handlings of the mAb is reduced from two to one.

The coordinative saturation of ^89^Zr in DFO* manifested itself also in vitro and in vivo. While the stability of ^89^Zr-DFO-trastuzumab with thiourea linker is strongly dependent on the storage conditions with respect to buffer composition, this is less critical for the thiourea-linked ^89^Zr-DFO*-trastuzumab. In previous experiments, we found that ^89^Zr-DFO-mAbs having a thiourea linker between DFO and the mAb should preferably not be stored in sodium chloride-containing buffers. We postulated that the presence of Cl^−^ ions affected the stability of ^89^Zr-labeled mAb due to radiation-induced formation of hypochlorite ions reacting with the enolised thiourea unit. Mixing of ^89^Zr-DFO-trastuzumab stored in 0.9 % NaCl with serum resulted in an increased stability, most probably because sulphur atoms of the serum proteins act as scavenging matter for the hypochlorite. The results in Table 1 show that ^89^Zr-DFO*-mAb with the same thiourea linker is far less sensitive to the presence of Cl^−^. This indicates that in case of DFO-*p*Phe-NCS, the enolised sulphur atom of the thiourea unit is coordinated to ^89^Zr and subsequently oxidised by hypochlorite. These results suggest that the unsaturated coordination of ^89^Zr is causing the observed impaired stability of the ^89^Zr-DFO complex, which is not seen with the new chelator DFO*.

Although blood kinetics and tumor uptake in mice were similar for ^89^Zr-DFO*-trastuzumab and ^89^Zr-DFO-trastuzumab, ^89^Zr-DFO*-trastuzumab showed a significantly lower uptake in bone containing organs, skin, liver, spleen, and ileum than ^89^Zr-DFO-trastuzumab (Table 2). Actually, over time the bone uptake of ^89^Zr-DFO-trastuzumab increased, while the uptake of ^89^Zr-DFO*-trastuzumab decreased (see Figure S5). Thus, DFO* indeed provides a product that is more stable in vivo, since ^89^Zr becomes less easily released from the chelator. Recently, much effort has been put in developing improved bifunctional chelators for ^89^Zr [16, 19, 20, 23]. Focusing on the bifunctional chelators that coordinate ^89^Zr efficiently, four molecules have been reported. Derivatised fusarinine C (FSC) [20] a cyclic hexadentate chelator, has been tested in combination with a RGD-peptide. Only short-term in vivo experiments are reported, and the performance with longer circulating biologicals is not yet known. Next, there are two ^89^Zr-HOPO-trastuzumab complexes reported [16, 23]. Deri et al. reported on ^89^Zr-3,4,3-(LI-1,2-HOPO)-mAb [16]. Although less bone uptake was observed for ^89^Zr-3,4,3-(LI-1,2-HOPO)-trastuzumab compared to ^89^Zr-DFO-trastuzumab, the tumor uptake also decreased to half of the uptake observed with ^89^Zr-DFO-trastuzumab. Tinianow et al. reported on ^89^Zr-2,3-HOPO-trastuzumab [23]. In vitro serum stability, radiochemical purity of the ^89^Zr-labeled mAb complex and in vivo bone uptake were less favorable for ^89^Zr-2,3-HOPO-mAb compared to ^89^Zr-DFO-mAb. Finally, Boros et al. reported on a macrocycle-based hydroxamate chelator L5 [19]. Although in vitro stability of their ^89^Zr-chelator was similar to ^89^Zr-DFO, its bifunctional variant coupled to trastuzumab showed higher bone uptake and faster blood clearance than ^89^Zr-DFO-trastuzumab. Though a direct comparison of the described hexadentate/octadentate bifunctional chelators for ^89^Zr is difficult, indications are that DFO* is the best qualified with respect to tumor targeting and in vitro and in vivo stability.

**Table 2:** Biodistribution of ^89^Zr-DFO*-trastuzumab and ^89^Zr-DFO-trastuzumab in N87 tumor-bearing nude mice at 24, 72, and 144 h after administration

	24 hr						72 hr						144 hr					
	DFO*			DFO			DFO*			DFO			DFO*			DFO		
Blood	13.45	±	1.20	16.16	±	1.47	10.06	±	1.22	9.49	±	2.13	3.73	±	1.84	3.50	±	2.41
Tumor	27.81	±	7.44	26.23	±	4.31	47.70	±	5.52	35.54	±	4.08	32.59	±	11.95	29.06	±	8.66
Skin	5.37	±	0.75	6.31	±	0.89	3.80	±	0.34	5.51	±	0.73	1.88	±	0.43	2.71	±	0.64
Tongue	4.69	±	0.36	5.74	±	0.67	3.44	±	0.33	3.52	±	0.47	1.31	±	0.60	1.60	±	0.76
Sternum	2.10	±	0.24	2.79	±	0.39	1.72	±	0.16	2.97	±	0.15	0.92	±	0.16	3.33	±	0.32
Heart	3.41	±	0.34	4.45	±	0.58	2.82	±	0.49	2.90	±	0.56	1.07	±	0.35	1.25	±	0.41
Lung	5.41	±	0.48	6.73	±	1.28	4.43	±	0.62	4.52	±	0.83	2.18	±	0.88	2.32	±	0.87
Liver	4.47	±	1.07	5.11	±	1.26	4.05	±	0.63	7.27	±	0.35	3.17	±	0.82	6.43	±	1.64
Spleen	3.36	±	0.99	3.95	±	0.68	2.68	±	0.23	3.22	±	0.30	1.65	±	0.34	3.26	±	1.05
Kidney	3.43	±	0.48	4.41	±	0.70	3.51	±	0.78	5.08	±	0.81	2.75	±	0.42	3.35	±	0.62
Bladder	3.75	±	0.28	4.56	±	0.19	3.65	±	0.46	4.04	±	0.41	1.65	±	0.50	2.01	±	0.40
Muscle	1.90	±	0.13	2.23	±	0.17	1.34	±	0.24	1.44	±	0.05	0.46	±	0.17	0.65	±	0.27
Femur	1.43	±	0.08	2.15	±	0.34	1.21	±	0.06	2.54	±	0.25	0.78	±	0,.11	3.85	±	0.80
Colon	1.42	±	0.08	1.79	±	0.25	1.18	±	0.19	1.41	±	0.15	0.74	±	0.29	1.06	±	0.28
Ileum	1.48	±	0.21	2.03	±	0.28	1.09	±	0.09	1.57	±	0.20	0.60	±	0.10	1.34	±	0.70
Stomach	1.61	±	0.25	1.97	±	0.20	1.33	±	0.18	1.57	±	0.27	0.67	±	0.14	0.94	±	0.12
Knee	n.d.			n.d.			2.07	±	0.15	5.70	±	1.51	1.38	±	0.23	8.20	±	2.94

Total administered dose 100 μg. Mean (%ID/g) ± SD at each time point after injection (*n* = six animals per time point and conjugate)

## Conclusion

^89^Zr-DFO*-mAbs can be prepared analogously to ^89^Zr-DFO-mAbs resulting in radioimmunoconjugates with high radiochemical purity and optimal immunoreactivity. The in vitro serum stability of ^89^Zr-DFO*-trastuzumab outperformed ^89^Zr-DFO-trastuzumab. In vivo ^89^Zr-DFO*-trastuzumab showed comparable tumor targeting and blood kinetics as ^89^Zr-DFO-trastuzumab, but less uptake in bone-containing organs, skin, liver, spleen, and ileum. This makes DFO* a candidate successor of DFO for clinical ^89^Zr-immuno-PET.

## Electronic supplementary material

Below is the link to the electronic supplementary material.

ESM 1 (GIF 2821 kb)

ESM 2 (GIF 2802 kb)

ESM 3 (PDF 645 kb)
