# Supplementary figures and images for: Comparison of the octadentate bifunctional chelator DFO*-pPhe-NCS and the clinically used hexadentate bifunctional chelator DFO-pPhe-NCS for 89Zr-immuno-PET
Source: Eur J Nucl Med Mol Imaging. 2016 Aug 30;44(2):286–95. doi: 10.1007/s00259-016-3499-x (PMC5215071; doi:10.1007/s00259-016-3499-x)

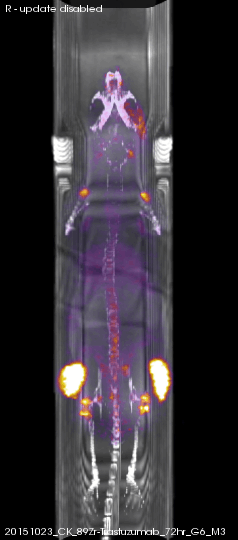

Supplement: Supplementary file 1 — (GIF 2821 kb) [file 259_2016_3499_MOESM1_ESM.gif]

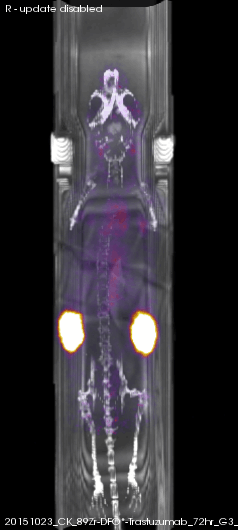

Supplement: Supplementary file 2 — (GIF 2802 kb) [file 259_2016_3499_MOESM2_ESM.gif]
