# Supplementary material for: Comparison of the octadentate bifunctional chelator DFO*-pPhe-NCS and the clinically used hexadentate bifunctional chelator DFO-pPhe-NCS for 89Zr-immuno-PET
Source: Eur J Nucl Med Mol Imaging. 2016 Aug 30;44(2):286–95. doi: 10.1007/s00259-016-3499-x (PMC5215071; doi:10.1007/s00259-016-3499-x)
Supplement: Supplementary file 3 — (PDF 645 kb) [file 259_2016_3499_MOESM3_ESM.pdf]

# Supplemental material to

## Comparison of the octadentate bifunctional chelator DFO*-*p*Phe-NCS and the clinically used hexadentate bifunctional chelator DFO-*p*Phe-NCS for $^{89}$Zr-immuno-PET

Danielle J. Vugts,[1] Chris Klaver,[1] Claudia Sewing,[1] Alex J. Poot,[1] Kevin Adamzek,[1] Seraina Huegli,[2] Cristina Mari,[2] Gerard W.M. Visser,[1] Ibai E. Valverde,[3] Gilles Gasser,[2] Thomas L. Mindt,[4,5] Guus A.M.S. van Dongen.[1]

[1] VU University Medical Center, Dept. of Radiology & Nuclear Medicine, Amsterdam, The Netherlands

[2] University of Zurich, Department of Chemistry, Zurich, Switzerland

[3] University of Basel Hospital, Division of Radiopharmaceutical Chemistry, Basel, Switzerland

[4] ETH Zurich, Institute of Pharmaceutical Sciences, Zurich, Switzerland

[5] Ludwig Boltzmann Institute for Applied Diagnostics, General Hospital of Vienna, Vienna, Austria

### Table of contents

General analyses 2

Synthesis of DFO*-*p*Phe-NCS 2

Fig. S1 $^{1}$H-NMR spectrum of DFO*-*p*Phe-NCS 3

Fig. S2 $^{13}$C-NMR spectrum of DFO*-*p*Phe-NCS 4

Fig. S3 HPLC spectrum of DFO*-*p*Phe-NCS at 275 nm 5

Fig. S4 HR-MS spectrum of DFO*-*p*Phe-NCS 6

Fig. S5 Uptake of $^{89}$Zr-DFO*-trastuzumab and $^{89}$Zr-DFO-trastuzumab in sternum, femur and knee at 24, 72 and 144 h p.i. 7

Fig. S6 Ratio of $^{89}$Zr-DFO-trastuzumab/$^{89}$Zr-DFO*-trastuzumab at 24, 72 and 144 h p.i. 8

Fig. S7 SE-HPLC chromatograms of $^{89}$Zr-DFO*-trastuzumab in 0.9% NaCl 9

Fig. S8 SE-HPLC chromatogram of $^{89}$Zr-DFO-trastuzumab in 0.9% NaCl 10

Table S1 *In vitro* stability of $^{89}$Zr-DFO*-trastuzumab and $^{89}$Zr-DFO-trastuzumab at RT 11

Summary of radiolabeling results and QC data of $^{89}$Zr-DFO*-cetuximab and $^{89}$Zr-DFO*-rituximab 12

**General analyses**

$^{1}$H-NMR and $^{13}$C-NMR spectra were measured with a Bruker ARX-400 or Bruker ARX-500 at room temperature. Chemical shifts (δ) are reported in ppm (parts per million) and coupling constant *J* in Hz. Residual solvent peaks were used as an internal reference. Abbreviations for the peak multiplicities are s (singlet), d (doublet), t (triplet), m (multiplet), br (broad). High resolution ESI-MS spectra were recorded using a Bruker ESQUIRE-LC quadrupole ion trap instrument. Ultra-performance liquid chromatography mass spectrometry (UPLC-MS) was performed with a Waters Acquity UPLC System with an Acquity UPLC PDA Detector and an Acquity UPLC BEH $C_{18}$ 1.7 µm reverse phase column (2.1 x 50 mm); flow rate: 0.6 mL/min with a gradient of A ($H_2O$ containing 0.1% formic acid) and B (acetonitrile): t = 0 min, 5% B; t = 0.5 min, 5% B; t = 4 min, 100% B; t = 5 min, 100% B. DFO*-*p*Phe-NCS was purified by preparative HPLC on a Phenomenex Jupiter 4u Proteo 90A (21.2 x 250 mm) column at a flow rate of 20 mL/min with a gradient of A (acetonitrile; Sigma-Aldrich HPLC-grade) and B (distilled water containing 0.1% TFA): t=0 min, A 5%; t=12 min, A 15%; t=24 min, A 35%; t=26 min, A 100%; t=28 min, A 100%; t=30 min, A 15%.

**Synthesis of DFO*-*p*Phe-NCS**

DFO* (50 mg, 0.066 mmol) was dissolved in a mixture of *i*-PropOH/$H_2O$ (4:1; 8 mL) and added dropwise to a solution of *p*-phenylenediisothiocyanate (126 mg, 0.66 mmol, 10 eq) and $Et_3N$ (11 µL, 0.079 mmol) in $CHCl_3$ (8 mL). The reaction mixture was stirred at room temperature for 24 h, concentrated under reduced pressure and the resulting white solid was washed with $Et_2O$ (4 x 10 mL). The product was purified by preparative HPLC to provide, after lyophilization, a white powder (6 mg, 10%, purity according to UPLC-MS at 275 nm >95%).$^{1}$H-NMR (600 MHz, DMSO-d6): δ(ppm) 9.64 (br m, 5 H), 7.90 (br s, 1 H), 7.77 (br s, 3 H), 7.54 (d, *J* = 12 Hz, 2 H), 7.36 (d, *J* = 6 Hz, 2 H), 3.41-3.49 (m, 10 H), 2.97-3.03 (m, 6 H), 2.55-2.60 (m, 6 H), 2.23-2.3 (m, 6 H), 1.97 (s, 3 H), 1.48-1.51 (m, 10 H), 1.36-1.39 (m, 6 H), 1.19-1.21 (m, 8 H). $^{13}$C-NMR (125 MHz, DMSO-d6): δ(ppm) 180.1, 172.0, 171.3, 170.2, 139.3, 132.6, 126.2, 124.6, 123.1, 47.1, 46.8, 43.7, 38.4, 29.9, 28.8, 28.0, 27.6, 27.1, 26.0, 23.6, 23.5, 20.4. Some carbon signals were not observed due to overlapping signals. HR-ESI-MS: calcd for $C_{42}H_{68}N_{10}O_{11}S_2$ $[M+H^+]^+$ 953.45887, found 953.45832.

The moderate yield of isolated DFO*-pPhe-NCS does not reflect low conversion of substrates or reagents but the challenging purification by HPLC due to low solubility of the compound in water and most organic solvents.

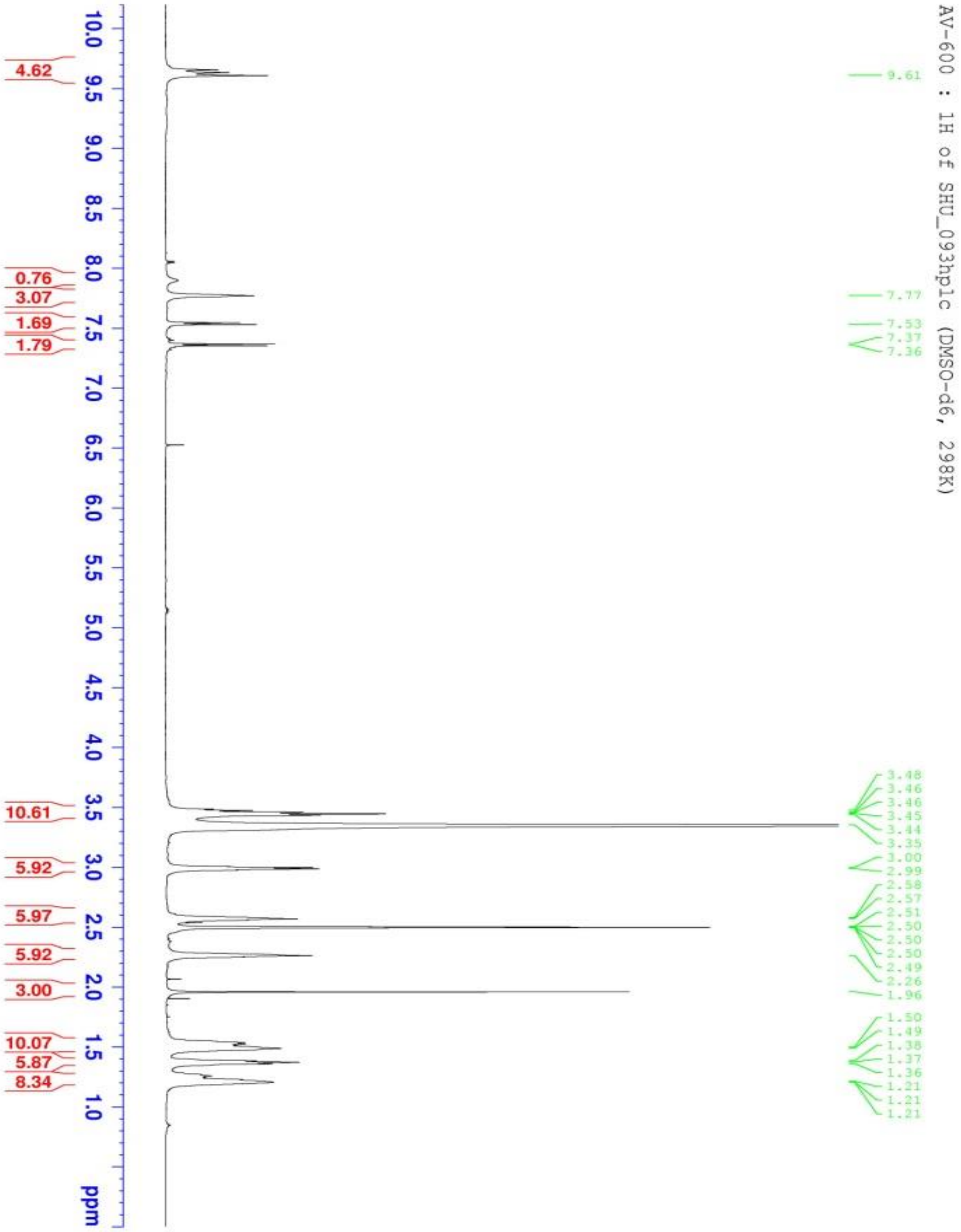


**Fig. S1** $^{1}$H-NMR spectrum of DFO*-*p*Phe-NCS

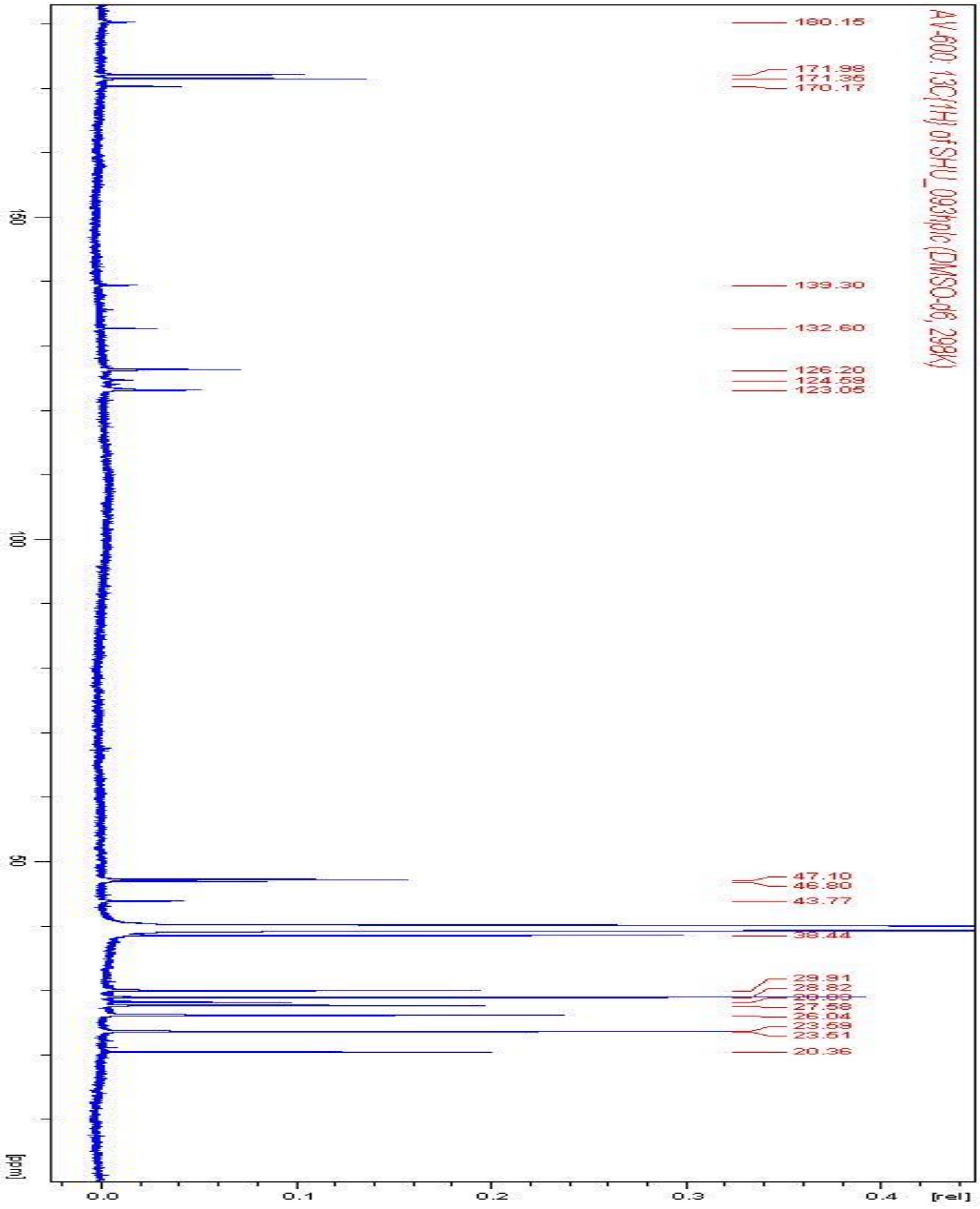


**Fig. S2** $^{13}$C-NMR spectrum of DFO*-*p*Phe-NCS

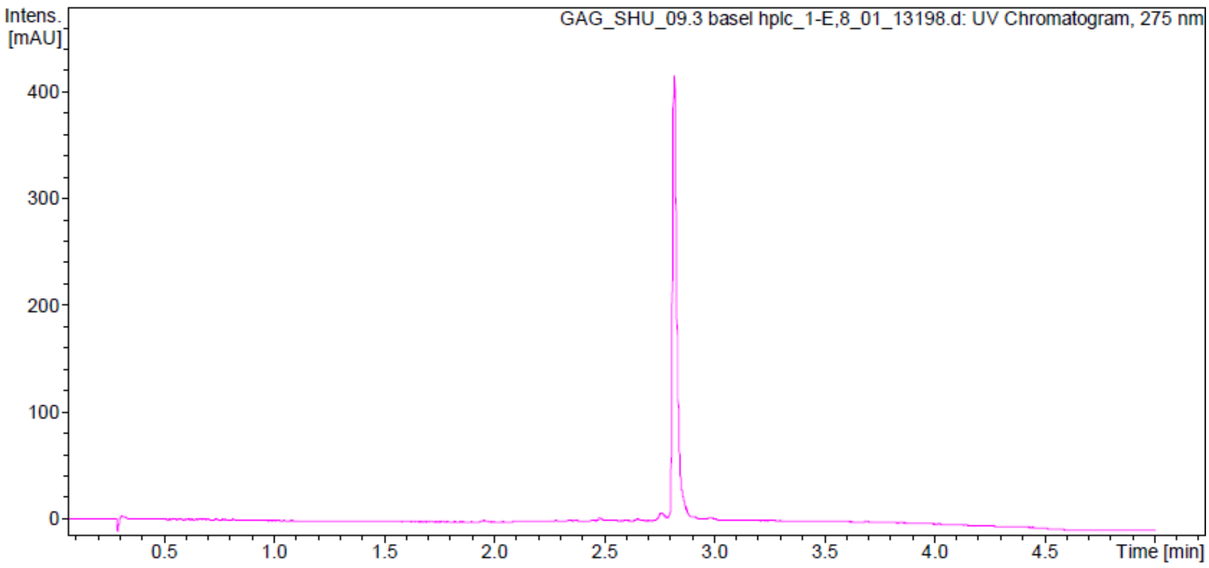


**Fig. S3** HPLC analysis of DFO*-*p*Phe-NCS at 275 nm

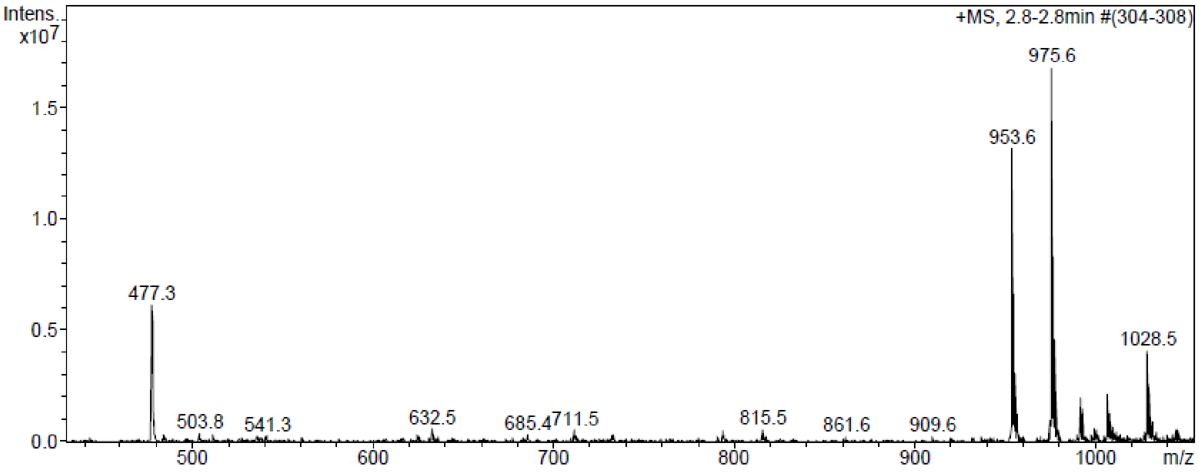


HR-ESI calcd for C42H68N10O11S2/*z* $[M+H^+]^+$ 953.45887, found 953.45832, calcd for

C42H68N10O11NaS2/*z* $[M+Na^+]^+$ 975.44027, found 975.44027

**Fig. S4** HR-MS spectrum of DFO*-*p*Phe-NCS

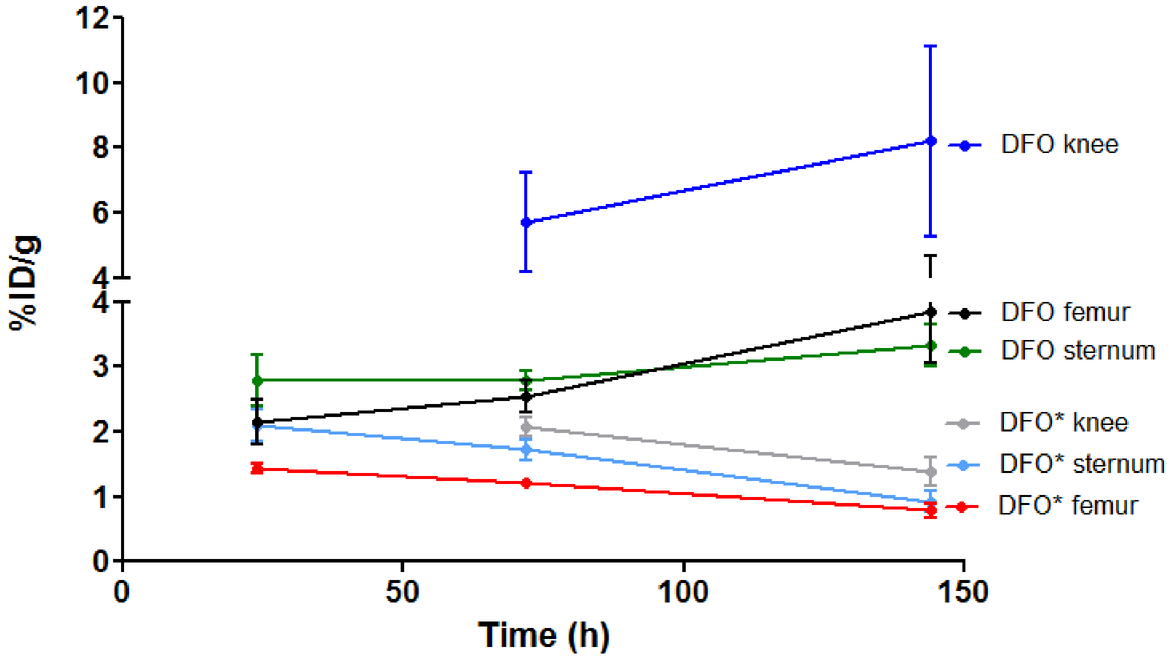


**Fig. S5** %ID/g in sternum, femur and knee for $^{89}$Zr-DFO*-trastuzumab and $^{89}$Zr-DFO-trastuzumab over time.

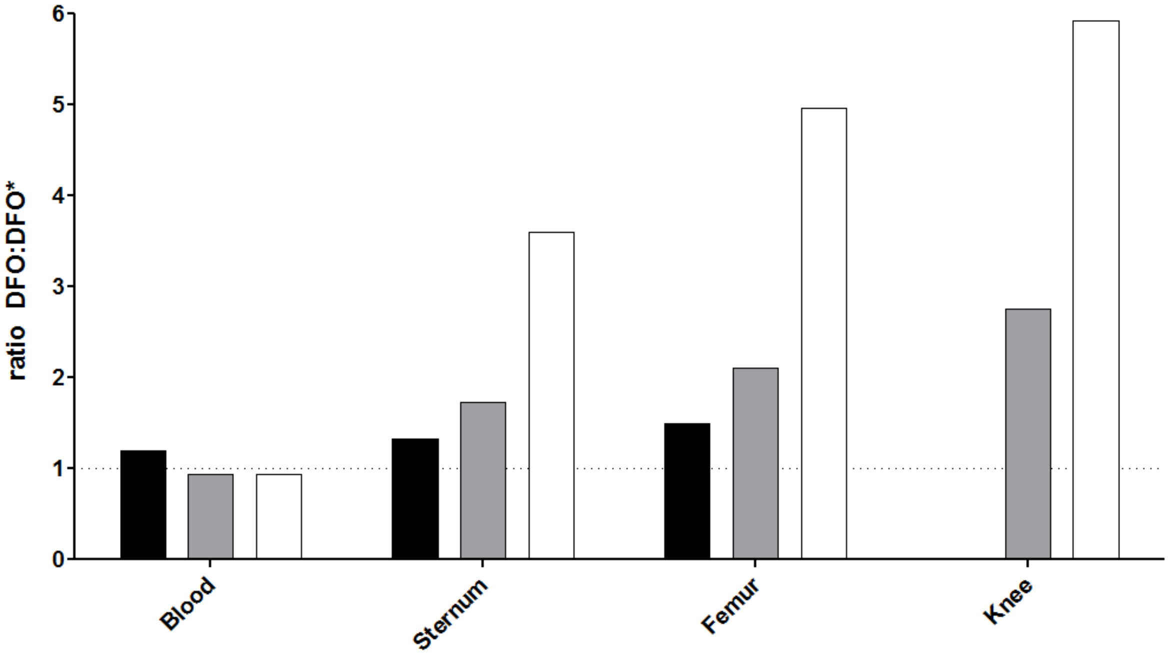


**Fig. S6** Ratio of $^{89}$Zr-DFO-trastuzumab/$^{89}$Zr-DFO*-trastuzumab in N87 tumor bearing nude mice at 24 h (black bar); 72 h (grey bar) and 144 h (white bar) after administration in blood, sternum, femur and knee.

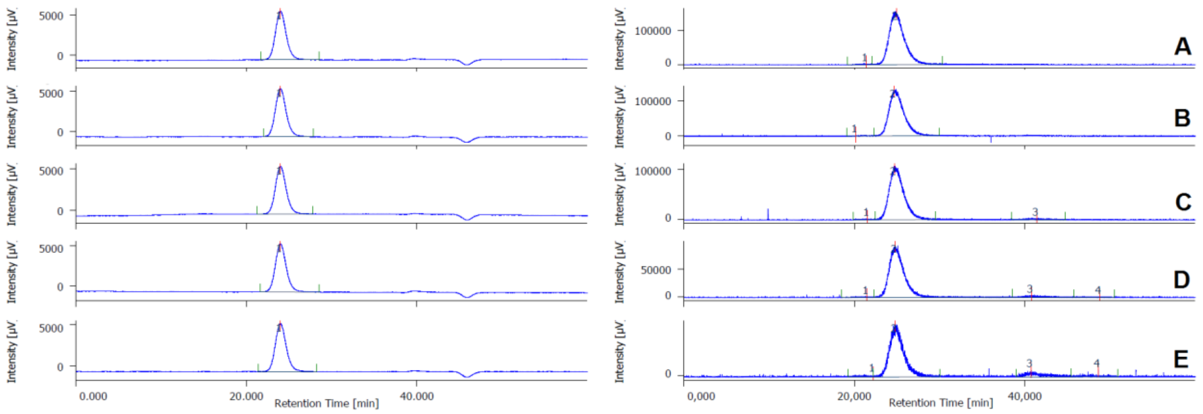


**Fig. S7** SE-HPLC chromatogram of $^{89}$Zr-DFO*-trastuzumab directly after formulation (A) and after storage at 2-8˚C for 24 h (B), 48 h (C), 72 h (D) and 168 h (E) in 0.9% NaCl. Left: UV at 280 nm and right: radioactive read-out.

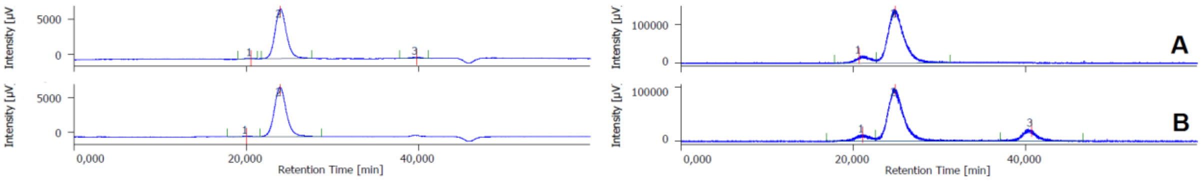


**Fig. S8** SE-HPLC chromatogram of $^{89}$Zr-DFO-trastuzumab directly after formulation (A) and after storage at 2-8˚C for 24 h (B) in 0.9% NaCl. Left: UV at 280 nm and right: radioactive read-out.

**Table S1** *In vitro* stability of $^{89}$Zr-DFO*-trastuzumab (A) and $^{89}$Zr-DFO-trastuzumab (B) in 20 mM histidine+ 240 mM sucrose and 0.9% NaCl at room temperature

| A | 20 mM Histidine/240 mM sucrose | | 0.9% NaCl | |
|---|---|---|---|---|
| | 21°C | | 21°C | |
| | Radiochemical purity (%) | Immunoreactive fraction (%) | Radiochemical purity (%) | Immunoreactive fraction (%) |
| 0 h | 99.0 | 98 | 98.0 | 97 |
| 24 h | 98.3 | 96 | 97.3 | 95 |
| 48 h | 96.8 | 95 | 93.5 | 91 |
| 72 h | 94.7 | 93 | 90.7 | 87 |
| 168 h | 84.1 | 81 | 76.5 | 72 |
| | | | | |
| B | 20 mM Histidine/240 mM sucrose | | 0.9% NaCl | |
| | 21°C | | 21°C | |
| | Radiochemical purity (%) | Immunoreactive fraction (%) | Radiochemical purity (%) | Immunoreactive fraction (%) |
| 0 h | 97.8 | 96 | 98.6 | 97 |
| 24 h | 96.5 | 94 | 75.2 | nd |
| 48 h | 95.6 | 92 | 57.3 | nd |
| 72 h | 92.9 | 92 | 41.1 | nd |
| 168 h | 87.5 | 85 | 42.7 | nd |

nd = not determined

Radiochemical purity of buffer samples determined by spin filter.

Radiochemical purity of serum samples determined by SEC-HPLC.

**Summary of radiolabeling results and QC data of $^{89}$Zr-DFO*-cetuximab and rituximab**

<u>$^{89}$Zr-DFO*-cetuximab</u>

Radiolabeling efficiency: 87%

Radiochemical purity (HPLC): 100%

(spin filter): 99.7%

Immunoreactive fraction: 99% (A431 fixated cells)

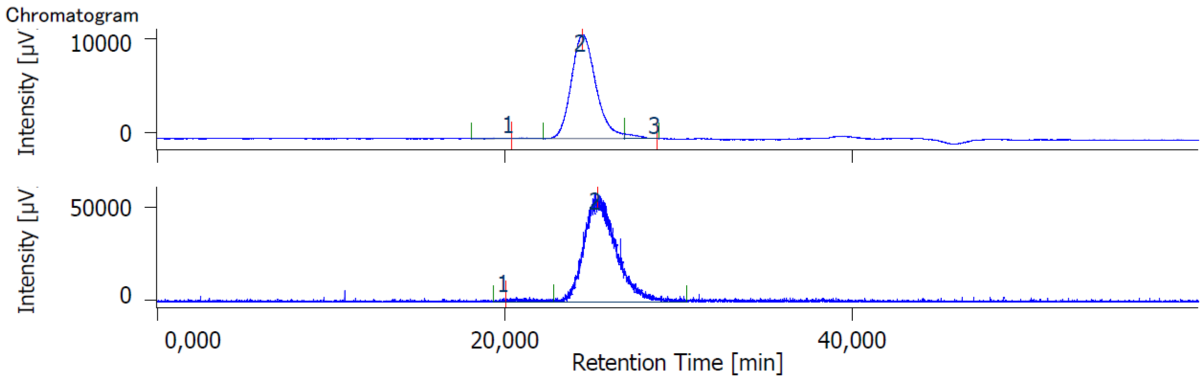


**Fig. S9** SE-HPLC chromatogram of $^{89}$Zr-DFO*-rituximab. Top: UV at 280 nm and bottom: radioactive read-out.

<u>$^{89}$Zr-DFO*-rituximab</u>

Radiolabeling efficiency: 89%

Radiochemical purity (HPLC): 100%

(spin filter): 99.4%

Immunoreactive fraction: 90% (SU-DHL4 fixated cells)

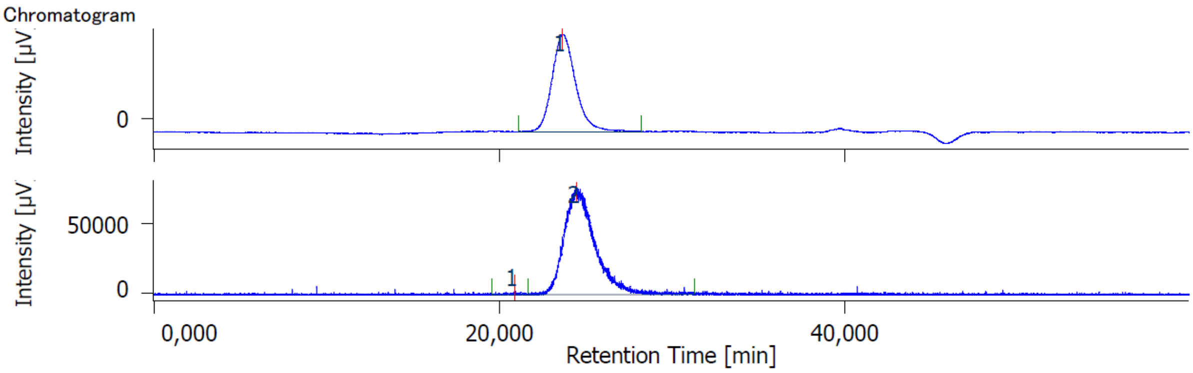


**Fig. S10** SE-HPLC chromatogram of $^{89}$Zr-DFO*-cetuximab. Top: UV at 280 nm and bottom: radioactive read-out.
